# Analyzing the advantages of subcutaneous over transcutaneous electrical stimulation for activating brainwaves

**DOI:** 10.1038/s41598-020-64378-6

**Published:** 2020-04-30

**Authors:** Wonok Kang, Jiho Lee, Yu Ri Kim, Woo Ram Chung, Duk L. Na, Young-Min Shon, Sung-Min Park

**Affiliations:** 1 School of Interdisciplinary Bioscience and Bioengineering, Pohang University of Science and Technology, Pohang, 37673 Republic of Korea; 2 Department of Creative IT Engineering, Pohang University of Science and Technology, Pohang, 37673 Republic of Korea; 3 Department of Neurology, Samsung Medical Center, Sungkyunkwan University School of Medicine, Seoul, 06351 Republic of Korea

**Keywords:** Neural circuits, Disorders of consciousness

## Abstract

Transcranial electrical stimulation (TES) is a widely accepted neuromodulation modality for treating brain disorders. However, its clinical efficacy is fundamentally limited due to the current shunting effect of the scalp and safety issues. A newer electrical stimulation technique called subcutaneous electrical stimulation (SES) promises to overcome the limitations of TES by applying currents directly at the site of the disorder through the skull. While SES seems promising, the electrophysiological effect of SES compared to TES is still unknown, thus limiting its broader application. Here we comprehensively analyze the SES and TES to demonstrate the effectiveness and advantages of SES. Beagles were bilaterally implanted with subdural strips for intracranial electroencephalography and electric field recording. For the intracerebral electric field prediction, we designed a 3D electromagnetic simulation framework and simulated TES and SES. In the beagle model, SES induces three to four-fold larger cerebral electric fields compared to TES, and significant changes in power ratio of brainwaves were observed only in SES. Our prediction framework suggests that the field penetration of SES would be several-fold larger than TES in human brains. These results demonstrate that the SES would significantly enhance the neuromodulatory effects compared to conventional TES and overcome the TES limitations.

## Introduction

Electrical stimulation is an efficacious therapeutic modality for neurological disorders, such as stroke, depression, and schizophrenia^1–5^. In this system, an electrical stimulus is delivered either invasively, e.g., deep brain stimulation (DBS), or non-invasively, e.g., transcranial magnetic stimulation (TMS), or by transcranial electrical stimulation (TES). Among these various stimulation methods, the TES technique has recently gained particular interest due to its ease of use, non-invasiveness, and cost-effectiveness. For modulating brain activity, TES induces electric fields in the brain by applying an electric current through the electrodes attached to the scalp. Based on the intended use of TES, different types of electrical current can be used to generates electric fields, e.g., direct current (transcranial direct current stimulation; tDCS), alternating current (transcranial alternating current stimulation; tACS) or pulsed current (transcranial pulsed current stimulation; tPCS), and so on. Among these various types of TES, research on tDCS and tACS is very active. The tDCS generally applies weak direct currents between two sponge electrodes placed on the scalp to either activate or suppress neurons in the brain^6–9^. The tACS delivers sinusoidal currents in a wide frequency range, i.e., 0.1 Hz to 200 kHz, without DC offset, which interferes with the cortical oscillatory activity or selectively modulates the neuronal membrane excitability^10–13^.

Numerous studies have been conducted to reveal issues related to the neuromodulatory effectiveness of TES, such as the field penetration ratio by shunting effects and allowable maximum current level to limit tissue damage. A study using an electromagnetic (EM) computational technique predicted that 2 mA TES could generate 0.8 mV/mm cortical electric fields^14^, and another study in movement disorders patients, implanted with DBS electrodes, reported that 4 mA bitemporal tDCS could generate about 0.26 mV/mm in deep brain area^15^. However, a recent study using human cadavers and rodents reported that an electric field >1 mV/mm is needed in rodent brains to directly induce the neuronal spikes^16^. By combining the recordings in rodents and human cadavers, Vöröslakos*. et. al*.^16^, estimated that, when applied transcutaneously, approximately 6 mA of current, which is beyond the safety limit for tDCS^17,18^, is needed to generate sufficient electric field to modulate neuronal circuits in human brains. Thus, several questions are being asked and debated currently in the scientific community about TES, including its clinical effectiveness, safety, and its applicability in human or relatively large animal brains.

Recently we proposed a subcutaneous electrical stimulation (SES) device to overcome the limitations of conventional TES^19^. In SES, a miniaturized stimulation device is placed under the scalp, and it applies electrical currents to the skull in contrast to TES, where the current is applied to the scalp. In that study, our SES method showed initial promise in theory as well as in limited evaluation. However, extensive comparisons between SES and TES to evaluate the practicality, safety and clinical effectiveness of subcutaneous stimulation compared to transcutaneous stimulation are still unknown, thus limiting its broader application.

In this study, to demonstrate the superiority of SES over TES, we present a comprehensive study comparing cerebral electric fields and neuromodulatory effects induced by SES and TES through *in vivo* experiments and extensive numerical simulations. Our step-by-step approach to compare these two techniques is as follows. First, we confirm the intracranial electric field and brain activity in the beagle head while performing transcutaneous and subcutaneous alternating current stimulation with varying stimulus parameters. Second, we construct an EM computational model and validate the model by comparing the simulated intracerebral fields induced by TES and SES against the corresponding experimental results. Finally, we extend our prediction framework to the human model to compare the electric fields induced in the human brain during subcutaneous and transcutaneous stimulation. These comprehensive *in vivo* and *in silico* comparisons would be a reliable predictor of SES performance over TES. Once validated, the clinical utility of SES can be expanded extensively. Thus, we believe our study is very important in the field of electrical stimulation for treating neurological disorders and would spawn future research and studies for advancing the application as well as the device, e.g., miniaturization. In the next few sections, we will present our methods, study setup, experiment, results, and conclusion.

## Results

### Intracranial electric field induced by subcutaneous and transcutaneous stimulation

**Figure 1:**
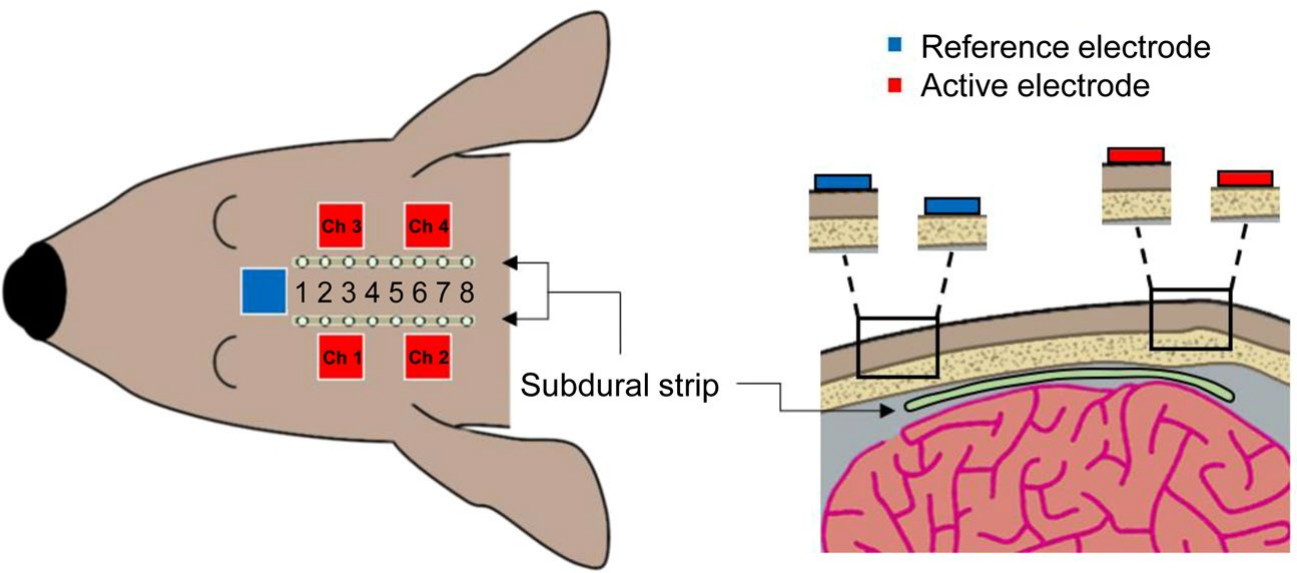
Schematic of the stimulating and recording electrode arrangement for subcutaneous and transcutaneous stimulation.

**Figure 2:**
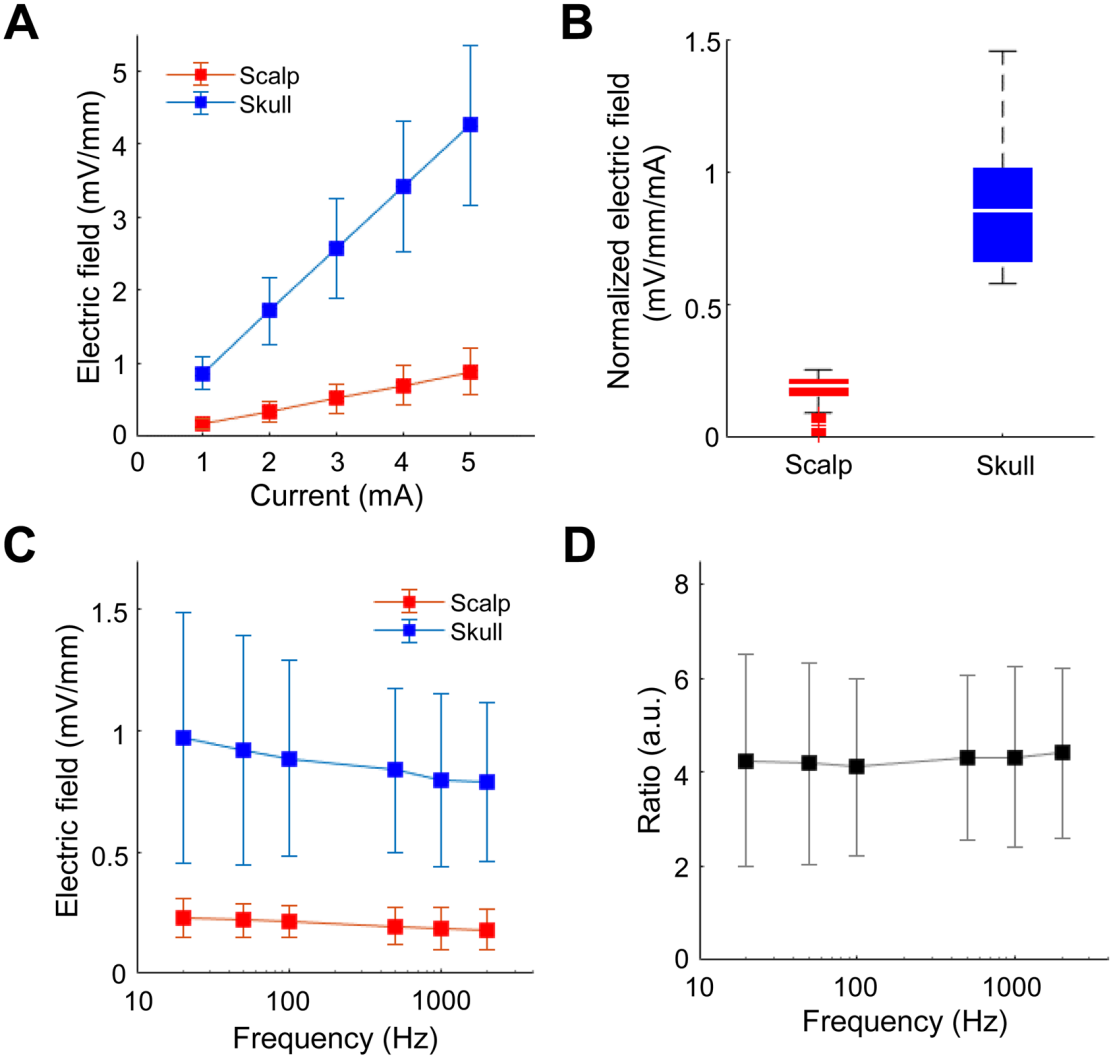
*In vivo* intracranial electric field induced by subcutaneous and transcutaneous stimulation. Measuring induced intracranial electric fields at the location of subdural strips. (**A**) Intracranial electric field induced by subcutaneous stimulation (R = 0.856, P < 0.001, n = 28 in four different arrangements in 3 beagles) was several times larger compared to transcutaneous stimulation (R = 0.760, P < 0.001, n = 28 in four different arrangements in 3 beagles). Error bars represent SD. (**B**) The ratio of induced intracranial electric field and stimulus intensity with subcutaneous and transcutaneous stimulation (P < 0.001, n = 140 in four different arrangements in 3 beagles). To induce 1 mV/mm intracranial electric field in transcutaneous stimulation, approximately 5 mA scalp-applied current is needed. (**C**) As the stimulus frequency from 20 to 2000 Hz increased, the induced field decreased by about 20% (n = 18 in two different arrangements in 3 beagles, R = −0.204, P = 0.034 for subcutaneous, and n = 20 in two different arrangements in 3 beagles, R = −0.279, P = 0.002 for transcutaneous stimulation). Error bars represent SD. (**D**) The ratio of intracranial gradients induced by subcutaneous and transcutaneous stimulation was almost constant with stimulus frequency between 20 and 2000 Hz (one-way ANOVA; F(5, 102) = 0.052, P = 0.998, n = 18 in 3 beagles for subcutaneous, and n = 20 in 3 beagles for transcutaneous stimulation). Error bars represent SD.

The intracranial electric fields induced by TES and SES techniques were measured to quantify the attenuation of the electric field via the scalp tissue using beagle models. Two 8-channel subdural strips were inserted between the skull and the brain (Fig. 1) through the drilled holes, and a needle electrode was inserted into the thigh to be served as the reference electrode for recording intracranial electric fields. The stimulation experiments were performed in two steps. In the first step, TES was performed. After TES, the scalp was surgically opened to perform SES. Five stainless steel stimulating electrodes with conductive electrode gel (four active electrodes and one reference electrode) were attached on the scalp or skull surface (Fig. 1). Stimulation currents with varying current intensity and frequency were applied sequentially to the four active electrodes, and the induced intracranial electric field was recorded. The comparisons between the magnitude of intracranial fields in the direction parallel to subdural strips induced by both TES (via scalp-applied current) and SES (via skull-applied current) techniques at the stimulus frequency of 1 kHz are shown in Fig. 2(A,B). Approximately 1.2 mA current was required to induce 1 mV/mm intracranial electric field in SES (Fig. 2A; blue line; Pearson’s linear correlation; R = 0.856, P < 0.001; n = 28), while approximately 5 mA current was needed to induce 1 mV/mm intracranial voltage gradient in TES (Fig. 2A; red line; Pearson’s linear correlation; R = 0.760, P < 0.001; n = 28). The subcutaneously applied current induced approximately four times larger intracranial electric fields compared to the transcutaneously applied current (Fig. 2B; 0.20 (IQR = 0.17–0.22) and 0.85 (IQR = 0.67–1.01) mV/mm/mA for TES and SES, respectively; paired *t*-test; P < 0.001; n = 140). These results imply the field in TES is attenuated due to the shunt effect in scalp tissue. The intracranial voltage gradients induced by subcutaneous and transcutaneous configurations with varying stimulus frequencies and fixed stimulus intensity at 1 mA are shown in Fig. 2(C,D). In both TES and SES, as the stimulus frequency increased from 20 to 2000 Hz, the induced intracranial field intensity decreased approximately by 20%; implying induced field intensity is related to stimulus frequency (Fig. 2C; Spearman’s rank correlation; R_subcutaneous_ = −0.204 and R_transcutaneous_ = −0.279; P_subcutaneous_ = 0.034 and P_transcutaneous_ = 0.002; n = 18 in 3 beagles for SES, and n = 20 in 3 beagles for TES). Between 20 and 2000 Hz stimulus frequency, the ratio between electric field induced by subcutaneously applied and transcutaneously applied current, E_subcutaneous_/E_transcutaneous_, was 4 and is independent of frequency (Fig. 2D; one-way ANOVA; P = 0.998; F(5, 102) = 0.052; n = 18 and 20 for subcutaneous and transcutaneous conditions, respectively). This finding confirms that the shunt effect in the scalp tissue was largely independent of the stimulus frequency. In addition, our findings were further supported by deriving the allowable range of the ratio of current penetration of SES to TES (Supplementary Figure 1).

**Figure 3:**
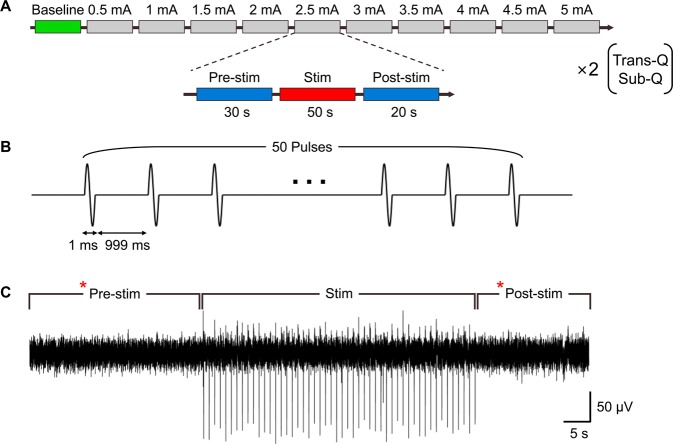
*In vivo* stimulation and iEEG recording protocol to confirm brain oscillations with transcutaneous and subcutaneous stimulation. Schematic overview of the stimulation protocol. (**A**) Experimental timeline showing stimulation sequence. This protocol was repeated equally for TES and SES. (**B**) Stimulus pattern for bi-phasic current stimulation. (**C**) Representative iEEG trace in one stimulation session. Asterisk (*) represents the data used for iEEG analysis.

**Figure 4:**
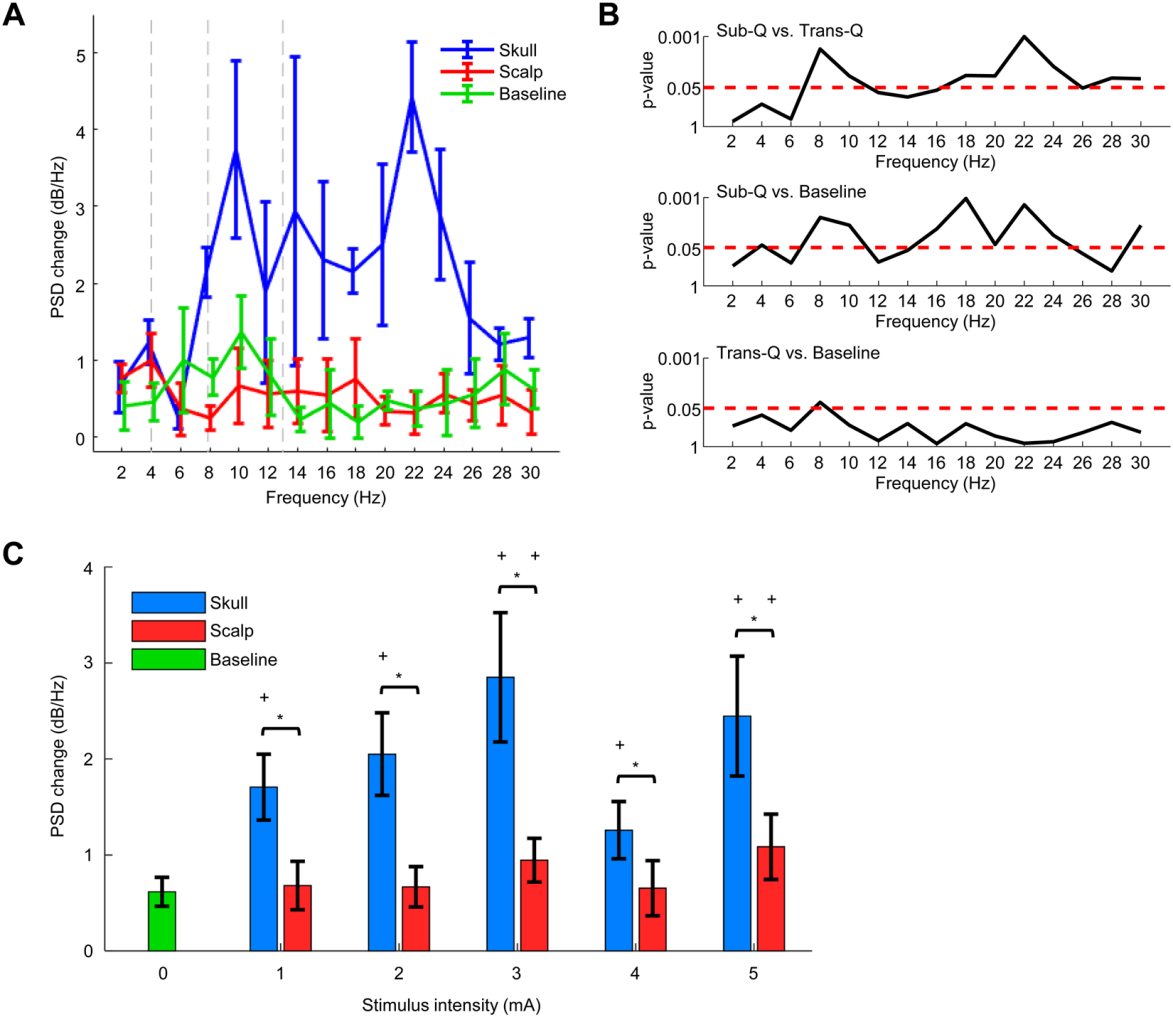
*In vivo* iEEG recording results from transcutaneous and subcutaneous stimulation. Comparison of transcutaneous and subcutaneous stimulation on the iEEG of a beagle. (**A**) Representative spectral power changes after transcutaneous (red line) and subcutaneous stimulation (blue line) in a frontal electrode cluster (postwave–prewave changes calculated with using a Hamming window with 50% overlap). PSD change between pre-stim and post-stim period was compared for different frequency bands (2–30 Hz, resolution of 2 Hz bins). (**B**) P-values show the difference in PSD changes between TES, SES, and baseline (n = 4, paired t-test). Skull-applied current induced iEEG changes in alpha and beta waves in contrast to scalp-applied current. (**C**) Changes in power in the frequency bands from delta to beta with varying stimulus intensity (mean ± CI, n = 64). SES induced PSD changes more than twice that of TES and baseline signal, largely independent of stimulus intensity. Asterisks code for significance (*, P < 0.01; +, P < 0.01 t-test with baseline signal). Delta (0.5–4 Hz), theta (4–8 Hz), alpha (8–13 Hz), and beta (13–30 Hz).

We tested the effects of scalp-applied and skull-applied current on the intracranial electroencephalogram (iEEG) activity in the beagle to make a direct physiological comparison. Figure 3 shows the overall stimulation protocol of the iEEG recording *in vivo*. The stimulus current (charge balanced sinusoidal pulse; Fig. 3B) was applied transcutaneously or subcutaneously using the custom made stimulator. The power spectral density (PSD) was computed for both TES and SES from the recorded signals in pre and post-stimulation sessions, at various stimulus intensities ranging from 0.5 mA to 5 mA with steps of 0.5 mA (Fig. 3A). The recorded waveforms of brain activity represented using dashed lines, as shown in Fig. 4A, are subdivided into four frequency bands for analysis: delta (0.5–4 Hz), theta (4–8 Hz), alpha (8–13 Hz), and beta (13–30 Hz). In SES, significant changes were found in alpha and beta waves in comparison to TES (Fig. 4B; PSD changes show significant differences against the baseline and TES conditions). To quantify iEEG changes against stimulus intensity, power changes from delta to beta bands were calculated (Fig. 4C; stimulus intensity from 0 to 5 mA, 1 mA each step). After transcutaneous stimulation, PSD with scalp-applied current did not change compared to baseline, implying this approach did not induce enough electric field to cause any changes in neuronal activity. In contrast, subcutaneous stimulation significantly changed PSD compared to baseline in all stimulus conditions, implying significant changes in neural activity.

**Figure 5:**
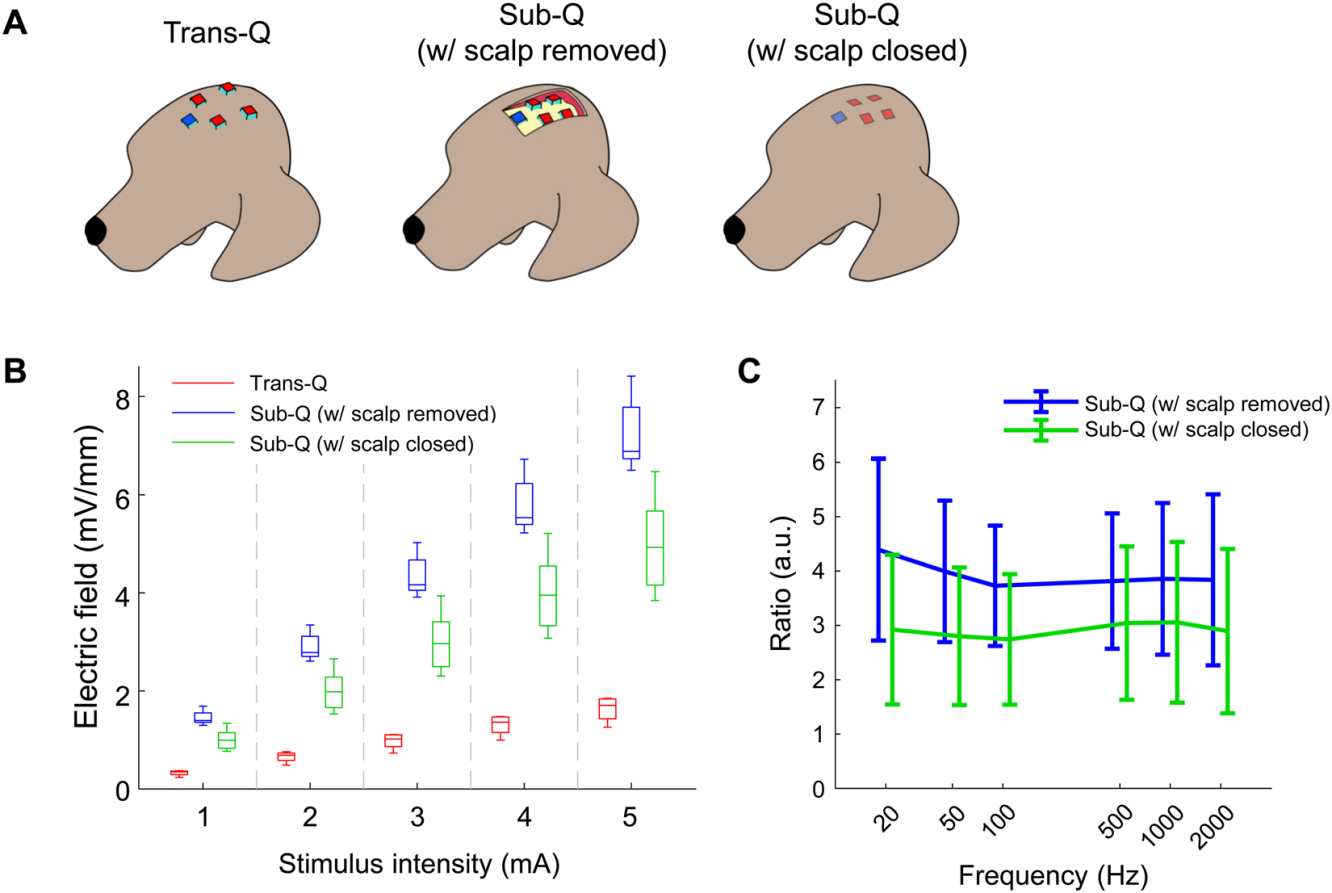
Possible changes in the shunting effect after stimulator implantation. Comparison of the intracranial electric fields induced by transcutaneous and subcutaneous stimulation (with scalp removed or closed). (**A**) Schematic of the electrode montage for each stimulation. (**B**) Electric fields induced by subcutaneous stimulation with scalp removed was approximately four-fold greater compared to transcutaneous stimulation (n = 12 in 2 beagles; 0.348, 0.687, 1.019, 1.362, and 1.704 mV/mm for TES; 1.395, 2.783, 4.166, 5.534, and 6.877 mV/mm for SES with scalp removed for 1, 2, 3, 4, and 5 mA intensities, respectively). This difference was reduced to about three times under condition where the scalp was completely closed (n = 12 in 2 beagles; 0.996, 1.981, 2.965, 3.953, and 4.928 mV/mm for SES with scalp closed for 1, 2, 3, 4, and 5 mA intensities, respectively). (**C**) In the frequency range from 20 to 2000 Hz, the ratio of induced voltage gradients of subcutaneous to transcutaneous stimulation decreased by about 25% due to the tissue covering the electrodes. Error bars represent SD.

The field and iEEG recordings of SES were performed with the skin tissues retracted and not closed after the electrode implantation. In other words, one side of the electrode was exposed to air and the other was placed in contact with the skull. This experimental configuration may lead to different results from human clinical conditions where restored tissues would fully cover the electrodes. To estimate changes in the level of shunting effects due to the tissue covering the stimulation device, we recorded intracranial voltage gradients induced by TES, SES with scalp removed, and SES with scalp closed (Fig. 5A). The field penetration of SES with scalp closed decreased by about 20–30% compared to the SES with scalp removed (Fig. 5B,C). These results show that subcutaneous stimulation induces electric fields approximately three times greater than the transcutaneous stimulation in real clinical scenarios.

In summary, *in vivo* experiments in beagles prove that the shunting effect in the scalp tissue attenuates a significant amount of transcutaneously applied current before reaching the cortical area. Independent of the stimulus frequency, approximately a quarter of the scalp-applied current is delivered to the brain in comparison to the skull-applied current. Our study clearly shows that the subcutaneous stimulation resulted in neuronal activation more effectively than the transcutaneous stimulation.

### Computational modeling to predict the intracerebral electric field

**Figure 6:**
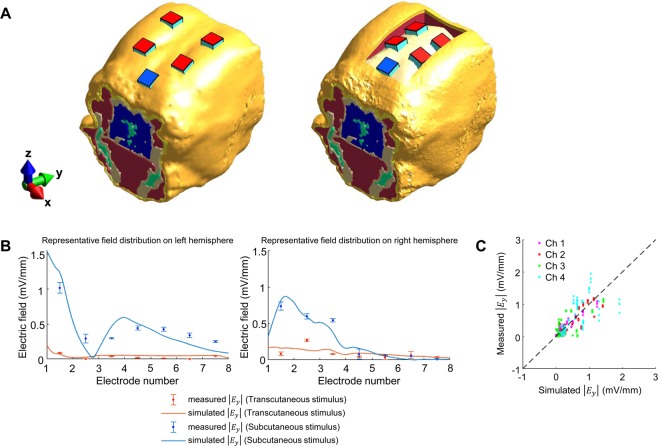
Validation of a computational 3D model to predict intracerebral fields. All values were measured and calculated in the y-direction. (**A**) EM computational 3D model for predicting electric fields induced by transcutaneous and subcutaneous stimulation. (**B**) In stimulation between the active electrode channel 1 and the reference electrode, the recorded values measured by using two-parallel subdural strips and the predicted values calculated by EM simulation were very similar to each other, and subcutaneous stimulation induced several times larger electric field compared to transcutaneous stimulation in EM simulation, too. Error bars represent SD. (**C**) The black dotted line means perfect electric field prediction. Our model showed very high accuracy of prediction (R = 0.788, P < 0.001).

**Figure 7:**
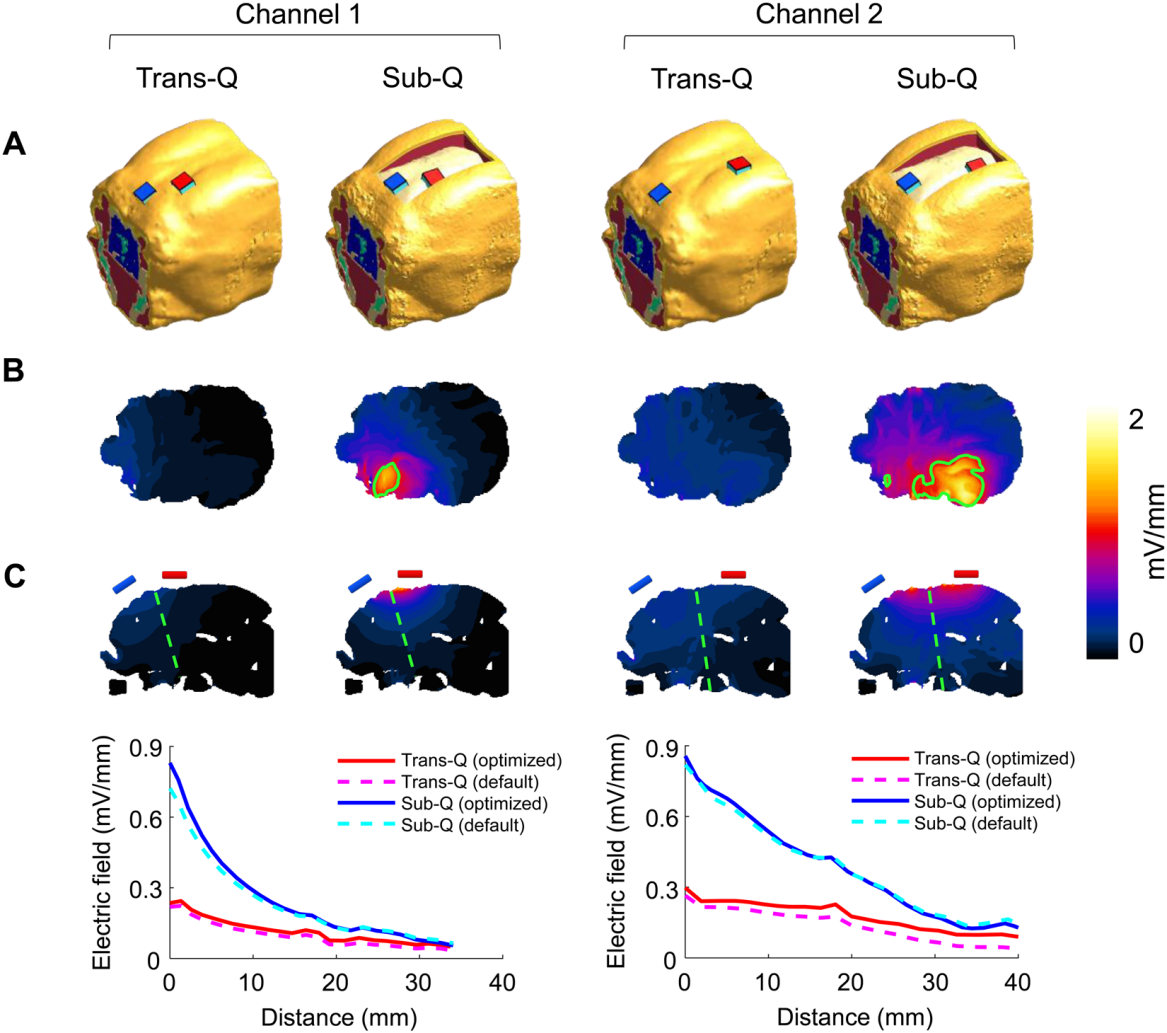
Prediction of intracerebral fields with the 3D model at 1 mA stimulus intensity and 1 kHz stimulus frequency. Comparing the prediction of intracerebral electric fields induced by transcutaneous and subcutaneous stimulation. (**A**) Two electrode placements, the same as those used in the *in-vivo* experiments (using channel 1 and 2 of the active electrodes and the reference electrode), were modeled for simulation. (**B**) Distribution map of the simulated electric fields in the transverse plane. The area where the fields are >1 mV/mm is surrounded by green boundary lines. (**C**) Showing predicted field intensity along the green dotted line that is perpendicular to the cortical surface. The voltage gradient decreased rapidly with increasing distance from the cortical surface in subcutaneous stimulation, whereas the field induced by transcutaneously applied current hardly changed. In all cases, the difference in field intensity was within 0.02 mV/mm on average estimated with the default or optimized tissue conductance.

The above-mentioned *in vivo* electric field recording technique measured the electric field induced on the surface of the brain and quantitatively demonstrated the superiority of SES over TES for current penetration. However, it could not measure the field formed outside the recording electrode. In addition, since the human response cannot be predicted based on the results from the experiments on a canine model, a translational method to extend the beagle study results to the real world human condition is necessary. Since this type of *in vivo* measurement in humans is difficult or can even be unsafe due to the invasiveness of the technique, we used a modeling framework commonly used in clinical studies to predict the electrical field in humans. Before making a prediction, the modeling framework should be validated, and thus we used *in vivo* beagle study results to validate our framework. The validation process and results are as follows. We designed a 3D EM simulation model based on magnetic resonance imaging (MRI) data of the 1^st^ beagle. Since there is very little existing literature reporting electrical conductivity values in canine or beagle tissue, we first applied the conductivities of human tissue reported in the existing literature^20–23^ and performed the EM simulation. Then we calibrated the model by adjusting conductivity values of scalp and skull tissues to minimize the difference and maximize the linear correlation coefficient between measured and simulated fields. The transcutaneous and subcutaneous stimulation setups were modeled with four active electrodes and one reference electrode (Fig. 6A). Simulations were performed with the stimulus intensity of 1 mA and frequency of 1 kHz, and the stimulation currents were sequentially applied to the four active electrodes, similar to *in vivo* experiments. The measured and simulated electric fields at two-parallel subdural strips are shown in Fig. 6(B,C). The voltage gradients along the recording electrodes in simulated and measured are similar to each other for both TES and SES techniques (Fig. 6B; representative results; stimulation between active electrode channel 1 and reference electrode). The strong correlation between measured and simulated results in Fig. 6C validates the capability of our numerical model to accurately predict the *in vivo* electric field. We also numerically demonstrated the prediction accuracy of the model (Fig. 6C) using Pearson’s correlation analysis (R = 0.788, P < 0.001, n = 128). Using our validated model, we performed a simulation to predict the induced electric field distribution and intensity with TES and SES in the beagle brain. The stimulation configurations are shown in Fig. 7A for predicting intracerebral electric fields in the beagle model with two electrode placements, similar to those used in *in vivo* experiments (stimulation between active electrodes channels 1 and 2 and reference electrode). In both electrode configurations, our model predicted that the 1 mA current applied subcutaneously is sufficient to induce >1 mV/mm intracerebral voltage gradients in the transverse plane, while the transcutaneously applied current does not induce sufficient field intensity anywhere in the brain at this current level (Fig. 7B). The electric field intensity in mid-brain areas varied with the depth from the cortical surface along the green dash line, as shown in Fig. 7C. The intracerebral electric field induced by the scalp-applied and skull-applied current decreased sharply with the increase in depth for both electrode arrangements.

Overall, our EM computational model predicted that the skull-applied current induces several-fold larger intracerebral electric fields near the cortex, compared to scalp-applied current. In the deep area, there was little difference in the induced voltage gradients when applied either subcutaneously or transcutaneously.

### Prediction of electric field induced by TES and SES in the human brain

**Figure 8:**
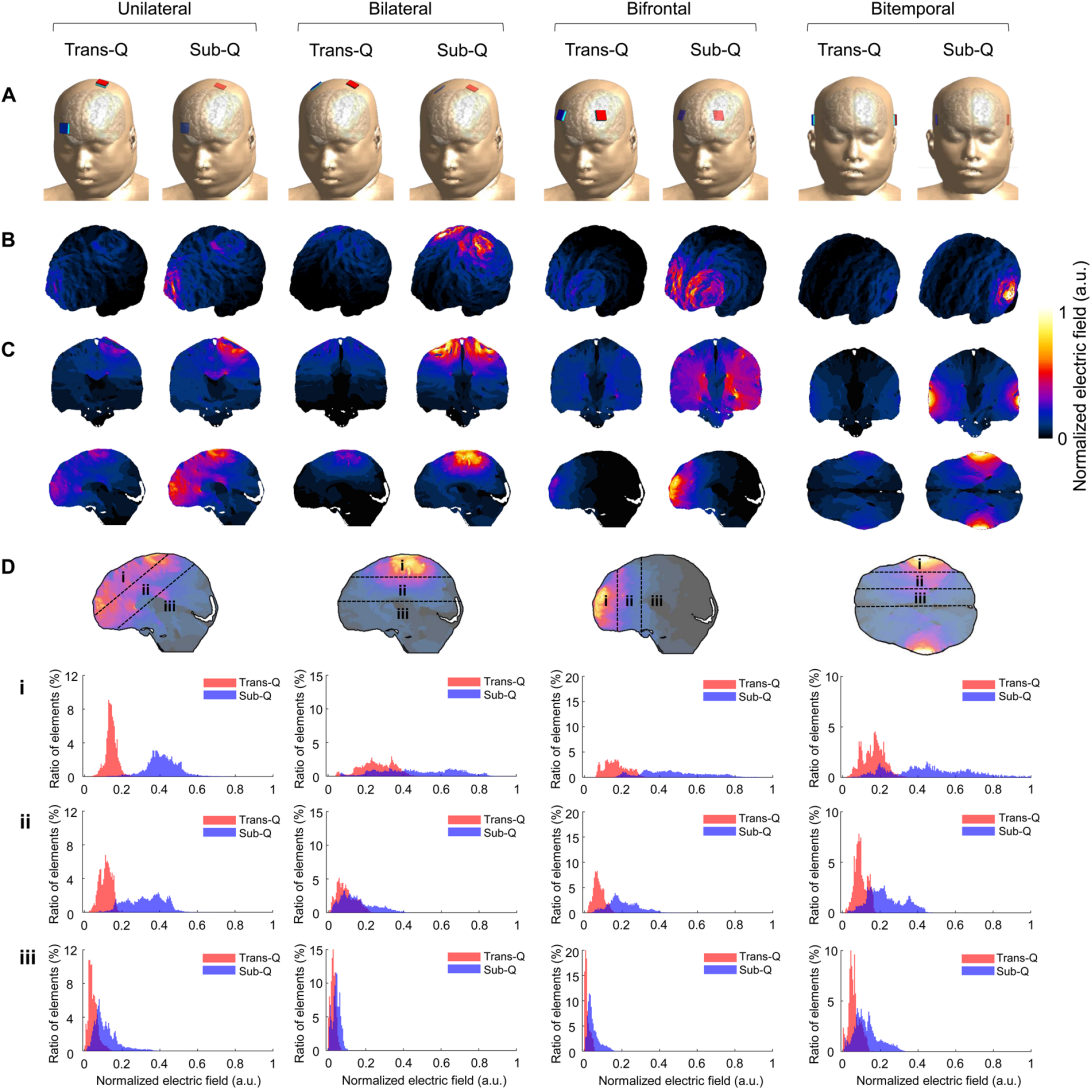
Comparison of induced electric fields at the same stimulus intensity and frequency in the human model. Predicting induced intracerebral electric fields in the human model. Field amplitudes were normalized to the maximum in subcutaneous condition for each case. (**A**) Various electrode montages for EM simulation to compare voltage gradients induced by transcutaneously applied and subcutaneously applied current. (**B**,**C**) Spatial distributions on cortical surface and cross-section plots show that obvious difference in cerebral fields induced by each placement. (**D**) As the distance from the stimulating electrodes increased, the difference in intracerebral electric field induced by subcutaneous and transcutaneous stimulation decreased.

**Figure 9:**
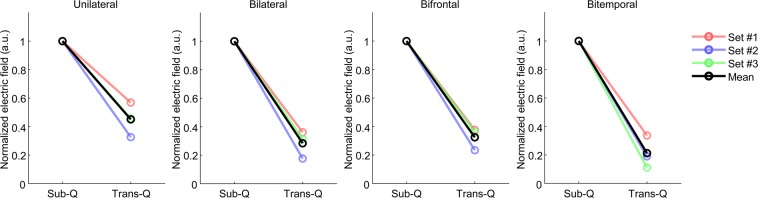
Prediction of TES and SES using various conductivity sets in four electrode montages. For various conductivity sets, the average ratio of induced voltage gradients of SES to TES was approximately 4. Field amplitudes were normalized to the average of the top 5% of induced fields in the whole brain volume in SES condition for each case.

Through both *in vivo* experiments and EM simulations with beagle models, we confirmed the superiority of the neuromodulatory effects of SES over the TES technique. To compare the electrical fields induced by TES and SES in the human brain, we extended our prediction framework to the human model that was developed recently for neuro studies^24–26^. The electrical conductivities of human tissue, reported in the literature^20–23^ and used in the EM simulation platform, were applied to the human model. Transcutaneous and subcutaneous setups were modeled with four electrode configurations, as shown in Fig. 8A. To observe and compare the electric fields generated by various electrode arrangements used in practical clinical settings, we modeled electrodes in unilateral, bilateral, bifrontal, and bitemporal placements. All stimulus configurations were simulated with the same current density and frequency. The normalized electric field amplitudes for each case are shown in Fig. 8(B,C). The maximum intensities of the voltage gradient were achieved at the cortical surface just under the stimulating electrodes (Fig. 8B,C). The gradual decrease in the induced fields with the increase in distance from the electrodes, when viewed from the sagittal (unilateral, bilateral, and bifrontal) or horizontal plane (bitemporal), is shown in Fig. 8C (bottom). Histograms of the electric field, for areas distanced uniformly from the stimulating electrodes, show that the subcutaneously applied current induced approximately four times larger intracerebral electric fields in comparison to transcutaneously applied current in all cases of distance (Fig. 8D). Since the shunting effect may depend on the electrical parameters of the tissues, we thus performed simulations with a total of three reported tissue parameters (Supplementary Table 1) to analyze changes in the shunting effect with various tissue conductivities. As the conductance of various tissues, including those of the scalp, skull, gray matter, and white matter, changed by several orders of magnitude, we found that in each montage there was an approximately two-fold difference between the highest and lowest estimated ratio of the induced electric field of SES to TES (Fig. 9). These results show that as the ratio of skin to bone conductivity increases, the level of shunting effects also increases.

In summary, our results with the human model predicted that a significant amount of energy is lost when the stimulation current was applied transcutaneously due to the shunting effect of scalp tissue. At any position in the brain, we found that the intensities of cerebral electric fields resulting from the SES would be several times greater than those from the TES.

## Discussion

Through intracranial electric field recordings using the beagle model, we found that the skull-applied current delivered several-fold greater stimulus to the cortical surface than the scalp-applied current. When the same current was applied in both TES and SES, the ratio of intracranial induced fields of TES to SES was almost constant within the measurement frequencies of 20 to 2000 Hz. From this finding, we believe that the current penetration ratio for both techniques will remain the same even at a frequency below 20 Hz, where low-frequency stimulations typically operate. Thus, independent of the stimulus method employed, such as tACS, tDCS, and tPCS, the stimulus current attenuates severely due to the shunting effect of skin and soft tissue. Consequently, to increase the neuromodulatory effects on brain circuits with transcutaneous stimulation methods, relatively large stimulus intensity would be required and thus at the risk of causing side effects such as tissue damage, dizziness, and phosphene perception^27–30^. On the contrary, the subcutaneous brain stimulation can provide neuromodulatory effects without these side effects since it is not affected by the shunting effect of the scalp.

Previous studies using rodents have shown that to induce measurable effects on neuronal spikes, a sufficient magnitude of charges should be injected through either the scalp or skull to achieve the threshold of 1 mV/mm at the target brain tissue^16,31,32^. However, to generate electric fields to consistently affect the neuronal network rhythms, several times more charges are required than those needed to induce action potentials^32–35^. By combining the findings from previous studies and our *in vivo* field recordings in this study, we believe that the subcutaneously applied current, even at a relatively low current level, can easily and effectively induce voltage gradients sufficient to derive neuromodulatory effects in beagle brain. As expected, we detected obvious changes in brain activity with recorded iEEG traces in subcutaneous stimulation. Changes in alpha and beta rhythms, which did not occur in transcutaneous stimulation, were manifested significantly in subcutaneous conditions. This suggests that the skull-applied current induced sufficient intracerebral electric fields to modulate local neuron networks in the *in vivo* experiments. In contrast, the scalp-applied current did not generate the voltage gradients above the thresholds needed to affect brain activity.

Computational methods have become accurate over the years^14,36–38^ and have been applied to predict real-world clinical cases^39–42^. In this study, we developed the modeling framework for comprehensively analyzing the difference between subcutaneous and transcutaneous stimulation. Our numerical model predicted that under the conditions of our *in vivo* experiments, the skull-applied current induces sufficient intracerebral electric fields to modulate neuronal circuits (>1 mV/mm), in contrast to scalp-applied current. These simulation results explain why only subcutaneous stimulation induced the change in brain activity, as shown in the iEEG recording, and this finding provides additional validation for the simulation technique adopted.

In building the electric field modeling framework, we assigned human tissue properties reported in the literature to our model. Then, based on the scientific rationale that living biological tissue properties can be different in each individual, we adjusted the conductivities to validate the framework within the specific ranges reported. Such a parametric optimization approach has been widely applied in modeling studies^14,43,44^. Our optimized conductivities of skull and scalp were approximately twice those of default values^20–23^ but were within the range reported in various literature^45–47^. The simulated electric fields using default or optimized values did not differ significantly. This result implies, the difference in dose-effect functions depends almost exclusively on different geometry or tissue thicknesses rather than on difference in tissue biophysics. Thus, we believe the optimization of tissue properties to validate the numerical EM model is a reasonable method and it can be widely used. However, for a fair comparison of all studies, the results using the reference values should be specified as a baseline.

Various clinical cases using human models were simulated using our prediction framework. As with the results of the beagle study, the simulation using the human model showed that in any stimulation arrangement, the charges applied transcutaneously are lost significantly before reaching the brain. On the other hand, the simulation predicted that subcutaneously applied current could deliver several times greater electric charges to the brain compared to the transcutaneously applied, and thus could result in significantly higher neuromodulatory effects. These results suggest that the SES, which can increase the stimulus delivered to the brain considerably than conventional TES, is a more powerful method to modulate neuronal activities.

In clinical TES, stimulus current is limited to less than 2 to 4 mA due to the potential side effects of high stimulation current intensity^17,18^. The tolerability of SES should also be considered since the stimulation device is implanted on the periosteum of the skull, connective tissue with relatively plenty of sensory fibers^48^. However, no report exists showing periosteal stimulation causing greater somatic or pain sensation than transcranial stimulation. In addition, SES requires only a quarter of current to achieve a similar performance as TES. Therefore, we believe SES is more immune to safety concerns, such as heating and pain sensation, compared to TES. Further parametric studies on stimulus intensity, shape, and duration will confirm the tolerability of SES.

Recently, studies have been conducted to not only stimulate cortex using TES but also for deep brain stimulation, and it is expected that this non-invasive approach could reduce the side effects of invasive open surgery (i.e., infections, intracranial hemorrhage, etc.)^49–51^. However, since our simulated results with beagle and human model showed that both scalp-applied and skull-applied current decreased drastically with the increase in depth from the cortical surface, we expect that neither TES nor SES can induce sufficient electric fields to directly stimulate the deep brain tissue. However, it may still be possible to stimulate the deep brain region, the cortex connected to the deep targets within the brain^52–54^ could be stimulated using SES technique. In addition, there are several promising novel approaches to focus the electric field onto the target region, including intersectional short pulse stimulation (ISP), temporal interference stimulation (TI), high definition-transcranial electrical stimulation (HD-TES)^16,50,55^. We expect that the combination of the technical concepts of the electric field focusing and the subcutaneous stimulation, discussed in this study, one could potentially enhance the direct effects of neuromodulation for the cortical region, thus achieving deep brain stimulation with a minimally invasive procedure.

While our study showed the superiority of SES over TES through comprehensive *in vivo* and *silico* study, a few additional studies could be performed to substantiate our results further. First, in our *in vivo* iEEG recording, only one set of pulse phase duration and repetition rate was used for stimulation. By performing a simulation with multiple pulse parameters, the results can be potentially generalized to other pulsed current stimulation parameters. Second, we compared TES and SES based on the generally accepted threshold level of neuronal activation (1 mV/mm) required to affect local networks within the living subject^16,56^. However, several *in vitro* studies have reported that neurons can be activated even in weak electric fields at the level of the sub-mV/mm^57,58^. Thus, our results cannot exclude the potential effects of the long-lasting low-intensity stimulation. By conducting parametric studies of stimulus intensity ranging from sub-mV to high levels for a long term period, the application area of SES can be expanded. Third, we minimized damage to the beagle’s tissue by maintaining the experimental condition similar to a normal state. Thus, space was limited for positioning electrodes for brain modulation. A study using optimal arrangements that elicit the largest drive on the neurons should be performed to further clarify the difference in neuromodulatory effects of SES to TES. Fourth, in human response prediction, we analyzed and compared the induced cerebral electric fields in normalized values because the human model used (in our EM prediction framework as well) was not validated with real *in vivo* data. However, we believe that the human response to electric field generation would be similar to the beagle model based on the results of previous simulation studies with the human model^14,15,59^.

In conclusion, we demonstrate that the subcutaneous stimulation induces several-fold greater cerebral electric fields compared to transcutaneous stimulation using *in vivo* experiments and extensive simulations. Thus, even with relatively low stimulus intensity, the skull-applied current may sufficiently modulate neuronal circuits in a human brain. While it can be contemplated that the subcutaneous electrodes may deliver more stimulation current to the target than transcutaneous conditions, to the best of our knowledge, ours is the first study to comprehensively analyze the SES and TES with *in vivo* and *silico* methods using a large animal model. Our findings propose a new paradigm of brain stimulation, which has lower invasiveness than intracranial stimulation and guarantees better neuromodulatory effectiveness than conventional TES techniques. In the near future, a miniaturized implantable subcutaneous brain stimulation device can be designed using technical advances, such as semiconductor, wireless power transfer, hermetic packaging technique, for minimally invasive procedures.

## Methods

### Ethical permissions

In this prospective study, all investigations were approved by the Institutional Animal Care and Use Committee at Samsung Medical Center in accordance with the handling of laboratory animals for biomedical research (approval number: 20180628003).

### MRI acquisition from the subjects

To make 3D EM model of the beagle, the three-dimension (3D) magnetization-prepared rapid gradient-echo (MP-RAGE) images of the beagle, under sedation with Telazol (5 mg/kg i.m.) and xylazine (2 mg/kg i.m.), were acquired using a Siemens 3.0 T Magnetom Prisma system with a 32-channel head coil. The following exam parameters were used: a repetition time of 2300 ms with an echo time of 3.4 ms, a slice thickness of 0.5 mm without slice interval, the flip angle of 8°, the field of view (FOV) of 150 mm, and slice number per slab of 224.

### Animals and surgical implantation of subdural electrodes

All dogs, weighing 11–13 kg, were initially sedated with Telazol (5 mg/kg i.m.) and xylazine (2 mg/kg i.m.). They were then intubated with an endotracheal tube in the prone position and were continuously ventilated with isoflurane (1%) under a standard stereotactic apparatus. A midline incision was performed to expose cranial landmarks - bregma and inion, to facilitate drilling of two burr holes on each hemisphere. Subsequently, two subdural strip electrodes (3 mm contact diameter, interelectrode interval 5 mm, 8 contacts; PMT Corporation, USA) were inserted parallel to the midline in a caudal-rostral direction. The cables (opposite to the electrodes) were rolled up and buried in a subcutaneous pocket made at the posterior neck of the dog. After filling the burr holes with medical bone cement to close the surgical incision, the location of the subdural strip electrodes was confirmed using fluoroscopic C-arm X-ray imaging.

### Design and performance of the custom made stimulator

Since the scalp has a very high impedance at low frequencies, high voltage is typically applied to stimulus via the scalp. A custom made current source device, which can drive voltages up to 70 V with an amplitude resolution of 4 µA and a frequency resolution of 0.1 Hz, was used for generating stimulation currents. To monitor the output current, a voltage drop across a 50 Ω resistor connected in series with the stimulating electrodes was measured by an instrumentation amplifier.

### Transcutaneous and subcutaneous stimulation-induced intracranial electric fields recording

After a 2-week recovery period, the beagles were re-intubated with an endotracheal tube and anesthetized with isoflurane (1.5–2%) in the prone position. After shaving the hair to expose the scalp completely, a 4 cm cut was made on the scalp behind the neck along the coronal plane for taking out the cables of two subdural strips. The cables were connected with NI-9220 (input impedance of 1 GΩ; common-mode rejection ratio of 70 dB; input range of ±30 V; 16-bit resolution; 16 analog input channels; maximum sampling rate of 100 kS/s/ch) data acquisition (DAQ) device (National Instruments, USA) using custom made connectors. The sample rate of DAQ was set to 20 kHz per channel, and no analog filter was used for data recording. A stainless steel electrode was inserted in the thigh for recording the reference. To electrically isolate the stimulating and recording system, they were powered using isolated AC/DC converter. Five stainless steel stimulation electrodes of 1 cm^2^ size each were configured with four active electrodes placed above the left and right hemispheres and one reference electrode placed above the frontal lobe. Analog switches were used to control the channels through which the stimulus current was applied. Transcutaneous stimulation was performed first with the electrode placement, as shown in Figs. 1 and 6A. The electrodes were attached to the scalp by conductive electrode gel. Transcutaneously applied currents, with varying intensities (1, 2, 3, 4, and 5 mA) at 1 kHz and varying frequencies (20, 50, 100, 500, 1000, and 2000 Hz) at 1 mA, were sequentially applied to the four active electrodes. The induced potentials were recorded for 3 seconds. After the transcutaneous stimulation experiment, the scalp was cut along the sagittal plane to expose the skull to perform subcutaneous stimulation. Electrodes were attached to the skull by conductive gel, as shown in Figs. 1 and 6A, and subcutaneous stimulation and potential measurement were performed under the same condition as transcutaneous stimulation. The recorded electric potentials were processed using MATLAB (MathWorks, USA) scripts to calculate the intracranial fields. Butterworth bandpass filter was used to eliminate noise, and filtered potentials were averaged before calculating the peak-to-peak amplitude. Finally, the magnitude of the electric field was derived by calculating the potential difference.

### iEEG analysis before and after stimulation to compare the efficacy of brain activation by transcutaneous and subcutaneous stimulation

After the dog was anesthetized with isoflurane (1.5–2%), the cables of subdural strip electrodes were pulled out through the small incision from the back of the neck. Since inhaling anesthetic agent would suppress the brain electrical activities resulting in intermittent iEEG recording, the continuous infusion of intravenous dexmedetomidine (0.3–0.7 µg/kg/hr) combined weak general anesthesia (maintained with 0.4–0.8% isoflurane) were used. This approach did not affect spontaneous interictal epileptiform activity, did not stimulate any additional motor activity, and also facilitated continuous iEEG recording^60–63^.

The iEEG recording was performed by the Nicolet ambulatory EEG system (Natus Medical Inc, USA) with 512 Hz sampling rates and subdural strip electrodes. A needle electrode inserted into the back of the dog’s neck served as the reference electrode for EEG machine. Two stainless steel electrodes of 1 cm^2^ size were attached to the skull with conductive gel; one electrode above the left hemisphere and the other electrode above the frontal lobe. The left hemisphere electrode served as the active electrode, and the frontal lobe electrode served as the reference for measurement. Stimulation and measurement protocols were used as follows; (1) Transcutaneous condition: After setting up the iEEG recordings, sufficient resting time was provided to stabilize the brain’s electrical activity. One stimulus session consisted of pre-stim (30 s), stim (50 s), and post-stim period (20 s), as shown in Fig. 3A. During one stim period, 50 stimulus pulses were delivered (1 Hz repetition rate, 0.5 ms pulse width bi-phasic, charge-balanced, and sinusoidal waveform; Fig. 3B), and stimulus current was sequentially varied from 0.5 mA to 5 mA with 0.5 mA step for each period (Fig. 3A). (2) Subcutaneous condition: Stimulation and iEEG recordings were performed with the same condition as transcutaneous stimulation. The recorded iEEG data were band-pass filtered between 1 to 100 Hz and notch filtered at 60 Hz to clean up the noise signal and then post-processed with MATLAB scripts to analyze the power spectrum of brain activity (using a Hamming window with 50% overlap).

### 3D beagle model construction

The 3D CAD model of a canine head was constructed from a 3D MRI image data (150 × 150 × 112 mm, 512 × 512 × 224 mm) and 2D C-Arm images (one sagittal and one transverse plane). The MRI data were transformed into DICOM format before importing it into the workplace. The 224 coronal planes were smoothed with a 6 × 6 average filter and were divided into small groups for the effective application of segmentation algorithms. Tissue segmentation was performed on iSeg platform (Zurich Med Tech AG, Switzerland). The segmentation for each tissue type in brain images went through several stages of image processing including morphological functions (dilating/eroding), interpolation, outline correction (OLC, removing speckles, filling holes, and so on). Tissues such as muscle, skull, gray matter, white matter, etc. were distinguished automatically using the gray level range, and error/outliers were corrected manually. Tissue segmentation information was imported into Sim4Life (Zurich Med Tech AG, Switzerland) and the surface of the 3D model was smoothed for visualization.

### Conductivity optimization and intracerebral field prediction for beagle model

To validate our model with *in vivo* recordings, we modeled the subdural strip and stimulating electrode by overlapping an X-ray image onto the 3D model. We first assigned conductivities of human tissue found in the literature to the model (in S/m): gray matter (0.0988); white matter (0.0626); cerebrospinal fluid (2); skull (0.00227); scalp (0.0002); blood (0.659); muscle (0.321); air (0); gel (0.6); stainless steel (1.45 × 10^6^)^20–23^. Next, we repeatedly performed simulations with 1 mA current and 1 kHz frequency for maximizing the correlation coefficient between the measured values and predicted values by adjusting conductivity values of scalp and skull within a range of values reported in various literature; (in S/m) skull: 0.0028–0.08^45^, scalp: 0.0002–1.0^21^. Finally, we found the conductivities of the skull (0.004 S/m) and scalp (0.0004 S/m) optimized for our model.

Simulations were performed using the ‘Quasi-static LF solver’ in Sim4Life with the sinusoidal stimulus of 1 mA, and 1 kHz applied sequentially to the two active electrodes (Fig. 7A). The data of all simulations were exported to MATLAB and were analyzed with custom-made scripts.

### Field computation with the human model

We modeled stimulating electrode configurations of subcutaneous and transcutaneous stimulation in the human model, Yoon-sun that was designed with magnetic resonance and computed tomography images for neuro studies^24–26^. For fair comparisons of TES and SES performance, the same size electrodes (4 cm^2^; gel (0.6 S/m); stainless steel (1.45 × 10^6^ S/m)) were used. Four electrode placements used in clinical trials were modeled; unilateral (F3-FP2), bilateral (F3-F4), bifrontal (FP1-FP2), bitemporal (T3–T4). Some of the conductivity values applied were similar to those applied to the beagle model ((in S/m) gray matter (0.0988); white matter (0.0626); cerebrospinal fluid (2); skull (0.00227); scalp (0.0002); blood (0.659); muscle (0.321); air (0); gel (0.6); stainless steel (1.45 × 10^6^))^21–23,37^ and the others were applied with the values, embedded in Sim4Life (Supplementary Table 1), provided by the IT’IS Foundation (Switzerland).

Simulations were performed with the current density of 0.25 mA/cm^2^ and sinusoidal stimulus frequency of 1 kHz, and the calculated cerebral fields were imported into the MATLAB for further calculations. For comparing predicted cerebral fields in the human model (Fig. 8), all field amplitudes were normalized to the maximum value of the field induced from each subcutaneous stimulation. To analyze the relationship between the shunting effect and tissue conductance parameter, three sets of conductivity reported in various literature (set #1: Gabriel’s literature values^20^, set #2 and #3: Huang’s literature and optimized values^14^; Supplementary Table 1) were applied to the model. All simulations were performed with the same stimulus intensity (0.25 mA/cm^2^) and frequency (1 kHz). All field intensities were normalized by the mean of the top 5% of the predicted values in the entire brain volume in subcutaneous stimulation (Fig. 9).

### Statistical analysis

Pearson’s linear correlation was calculated to analyze the linear correlation of data that follows a normal distribution, and Spearman’s rank correlation was calculated to analyze the monotonic relationship for data that do not follow the normal distribution. We also plotted the mean ± SD values for the entire data sets. Student’s paired *t*-test was used to compare pairwise data. We reported the median and interquartile range (IQR) for data that did not follow the normal distribution. To show the medians, interquartile ranges, and full ranges of the data, we used box-and-whisker plots. The one-way ANOVA with Bonferroni corrections was used to analyze the difference among group means in data. For simplicity, P values larger than 0.001 were reported in absolute value; otherwise, P values were reported as P < 0.001.

## Supplementary information

Supplementary information.
