# Supplementary material for: Analyzing the advantages of subcutaneous over transcutaneous electrical stimulation for activating brainwaves
Source: Sci Rep. 2020 Apr 30;10:7360. doi: 10.1038/s41598-020-64378-6 (PMC7193608; doi:10.1038/s41598-020-64378-6)
Supplement: Supplementary file 1 — Supplementary information. [file 41598_2020_64378_MOESM1_ESM.pdf]

Supplementary information

Title: Analyzing the advantages of subcutaneous over transcutaneous electrical stimulation for activating brainwaves

Authors: Wonok Kang[1], Jiho Lee[2], Yu Ri Kim[2], Woo Ram Chung[3], Duk L. Na[3], Young-Min Shon[3,**], Sung-Min Park[1,2,*]

[1] School of Interdisciplinary Bioscience and Bioengineering, Pohang University of Science and Technology, Pohang, 37673, Republic of Korea

[2] Department of Creative IT Engineering, Pohang University of Science and Technology, Pohang, 37673, Republic of Korea

[3] Department of Neurology, Samsung Medical Center, Sungkyunkwan University School of Medicine, Seoul, 06351, Republic of Korea

*** Corresponding author.**

Sung-Min Park, Ph.D.

Department of Creative IT Engineering, Pohang University of Science and Technology, Pohang, 37673, Republic of Korea.

E-mail: sungminpark@postech.ac.kr

**** Corresponding author.**

Young-Min Shon, MD, Ph.D.

Department of Neurology, Samsung Medical Center, Sungkyunkwan University School of Medicine, Seoul, 06351, Republic of Korea.

E-mail: youngmin.shon@samsung.com

$$I_{TES} = I_{applied}(1 - (R_{shunt} + R_{series}))$$

$$I_{SES} = I_{applied}(1 - R_{series})$$

$$\text{Ratio of } I_{SES} \text{ to } I_{TES} = \frac{1 - R_{series}}{1 - (R_{shunt} + R_{series})}$$

$$0.08 < R_{series} < 0.16, 0.45 < R_{shunt} < 0.65$$

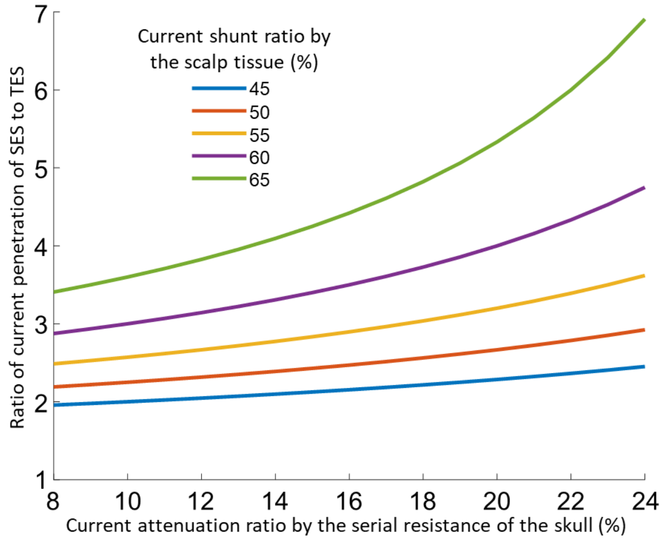

Supplementary Figure 1. Ratio of current penetration of SES to TES

The ratio of current penetration of SES to TES reaching the brain is from a minimum of 2 to a maximum of 7, based on literature data (Vöröslakos, Takeuchi et al. 2018). The larger the ratio of current shunt, the larger the change in penetration ratio according to the change in the ratio of current attenuation.

Supplementary Table 1. Tissue conductance parameters for the human model simulation (in S/m)

Material \ Set No.	Set #1 (used in Fig. 7 and 8)	Set #2 (used in Fig. 8)	Set #3 (used in Fig. 8)
Air	0	0	0
	(Gabriel 1996)		
Blood	0.7	0.7	0.7
	(Hahn, Kernahan et al. 1980, Gabriel 1996)		
Bone	0.0022655	0.01	0.03
	(Gabriel 1996)	(De Mercato and Sanchez 1992, Gabriel 1996, Akhtari, Bryant et al. 2002)	(De Mercato and Sanchez 1992, Gabriel 1996, Akhtari, Bryant et al. 2002, Huang, Liu et al. 2017)
Cartilage	0.17428	0.17428	0.17428
	(Gabriel 1996)		
Cerebellum	0.1188	0.1188	0.1188
	(Gabriel 1996)		
Cerebrospinal Fluid	2	1.65	1.65
	(Gabriel 1996, Baumann, Wozny et al. 1997)	(Freygang Jr and Landau 1955, Ranck Jr 1963, Gabriel 1996, Huang, Liu et al. 2017)	
Commissura Posterior	0.265076	0.265076	0.265076
	(Stoy, Foster et al. 1982, Gabriel 1996, Latikka, Kuurne et al. 2001)		
Dura	0.50076	0.50076	0.50076
	(Gabriel 1996)		
Eye	0.5048	0.5048	0.5048
	(Gabriel, Sheppard et al. 1983, Gabriel 1996)		
Fat	0.022404	0.022404	0.022404
	(Durney, Massoudi et al. 1986, Rigaud, Hamzaoui et al. 1994, Gabriel 1996)		
Gel	0.6	0.6	0.6
	(Armenean, Perrin et al. 2004)		
Gray Matter	0.098805	0.276	0.82
	(Stoy, Foster et al. 1982, Gabriel 1996)	(Freygang Jr and Landau 1955, Ranck Jr 1963, Gabriel 1996)	(Freygang Jr and Landau 1955, Ranck Jr 1963, Gabriel 1996, Huang, Liu et al. 2017)

Hippocampus	0.275634	0.275634	0.275634
	(VAN, Murphy et al. 1963, Stoy, Foster et al. 1982, Gabriel 1996, Latikka, Kuurne et al. 2001)		
Hypophysis	0.4811	0.4811	0.4811
	(Gabriel 1996)		
Hypothalamus	0.239149	0.239149	0.239149
	(VAN, Murphy et al. 1963, Stoy, Foster et al. 1982, Gabriel 1996, Latikka, Kuurne et al. 2001)		
Medulla Oblongata	0.234007	0.234007	0.234007
	(Freygang Jr and Landau 1955, VAN, Murphy et al. 1963, Geddes and Baker 1967)		
Midbrain	0.234007	0.234007	0.234007
	(Freygang Jr and Landau 1955, VAN, Murphy et al. 1963, Geddes and Baker 1967)		
Mucous Membrane	0.355287	0.355287	0.355287
	(Geddes and Baker 1967, Hahn, Kernahan et al. 1980, Stoy, Foster et al. 1982, Hart and Dunfee 1993, Gabriel 1996)		
Muscle	0.32115	0.32115	0.32115
	(Epstein and Foster 1983, Gielen, Wallinga-de Jonge et al. 1984, Durney, Massoudi et al. 1986, Hart and Dunfee 1993, Bodakian and Hart 1994, Gabriel 1996)		
Nerve	0.265076	0.265076	0.265076
	(Stoy, Foster et al. 1982, Gabriel 1996, Latikka, Kuurne et al. 2001)		
Pons	0.234007	0.234007	0.234007
	(Freygang Jr and Landau 1955, VAN, Murphy et al. 1963, Geddes and Baker 1967)		
Salivary Gland	0.67	0.67	0.67
	(Halter, Hartov et al. 2007)		
Skin	0.00020006	0.465	0.29
	(Yamamoto and Yamamoto 1976, Gabriel 1996)	(Burger and Van Milaan 1943, Hasted 1973, Gabriel 1996)	(Burger and Van Milaan 1943, Hasted 1973, Gabriel 1996, Huang, Liu et al. 2017)
Stainless steel	1450000	1450000	1450000
	(Elert 2011)		
Subcutaneous Fat	0.022404	0.022404	0.022404
	(Durney, Massoudi et al. 1986, Rigaud, Hamzaoui et al. 1994, Gabriel 1996)		
Thalamus	0.239149	0.239149	0.239149
	(VAN, Murphy et al. 1963, Stoy, Foster et al. 1982, Gabriel 1996, Latikka, Kuurne et al. 2001)		
Tongue	0.355287	0.355287	0.355287
	(Geddes and Baker 1967, Hahn, Kernahan et al. 1980, Stoy, Foster et al. 1982, Hart and Dunfee 1993, Gabriel 1996)		
Trachea	0.341987	0.341987	0.341987
	(Gabriel 1996)		
Trachea Lumen	0	0	0
	(Gabriel 1996)		
White Matter	0.062574	0.126	0.38
	(Stoy, Foster et al. 1982, Gabriel 1996)	(Freygang Jr and Landau 1955, Ranck Jr 1963, Gabriel 1996)	(Freygang Jr and Landau 1955, Ranck Jr 1963, Gabriel 1996, Huang, Liu et al. 2017)

Conductance values for the three parameter sets were determined based on the literature entitled “Compilation of the Dielectric Properties of Body Tissues at RF and Microwave Frequencies (Gabriel 1996)”. Tissue conductivities of parameter sets #2 and #3, including the scalp, skull, gray matter, white matter, and cerebrospinal fluid, were changed based on literature that validated current-flow models in humans (Huang, Liu et al. 2017).

References

Akhtari, M., et al. Conductivities of three-layer live human skull. Brain Topography 14, 151-167 (2002).

Armenean, C., et al. RF-induced temperature elevation along metallic wires in clinical magnetic resonance imaging: influence of diameter and length. Magnetic Resonance in Medicine 52, 1200-1206 (2004).

Baumann, S. B., et al. The electrical conductivity of human cerebrospinal fluid at body temperature. IEEE transactions on

biomedical engineering 44, 220-223 (1997).

Bodakian, B., et al. The dielectric properties of meat. IEEE Transactions on Dielectrics and Electrical Insulation 1, 181-187 (1994).

Burger, H., et al. Measurements of the specific resistance of the human body to direct current. Acta Medica Scandinavica 114, 584-607 (1943).

De Mercato, G., et al. Correlation between low-frequency electric conductivity and permittivity in the diaphysis of bovine femoral bone. IEEE transactions on biomedical engineering 39, 523-526 (1992).

Durney, C. H., et al. Radiofrequency radiation dosimetry handbook. (Utah Univ Salt Lake City Dept of Electrical Engineering, 1986).

Elert, G. Resistivity of steel. The physics factbook (2011).

Epstein, B., et al. Anisotropy in the dielectric properties of skeletal muscle. Medical and Biological Engineering and Computing 21, 51 (1983).

Freygang Jr, W., et al. Some relations between resistivity and electrical activity in the cerebral cortex of the cat. Journal of Cellular and Comparative Physiology 45, 377-392 (1955).

Gabriel, C. Compilation of the dielectric properties of body tissues at RF and microwave frequencies. (King's coll london (United Kingdom) dept of physics, 1996).

Gabriel, C., et al. Dielectric properties of ocular tissues at 37 degrees C. Physics in Medicine and Biology 28, 43 (1983).

Geddes, L. A., et al. The specific resistance of biological material—a compendium of data for the biomedical engineer and physiologist. Medical and biological engineering 5, 271-293 (1967).

Gielen, F., et al. Electrical conductivity of skeletal muscle tissue: Experimental results from different musclesin vivo. Medical and Biological Engineering and Computing 22, 569-577 (1984).

Hahn, G. M., et al. Some heat transfer problems associated with heating by ultrasound, microwaves, or radio frequency. Annals of the New York Academy of Sciences

335, 327-346 (1980).

Halter, R. J., et al. Electrical impedance spectroscopy of the human prostate. IEEE transactions on biomedical engineering 54, 1321-1327 (2007).

Hart, F., et al. In vivo measurement of the low-frequency dielectric spectra of frog skeletal muscle. Physics in Medicine and Biology 38, 1099 (1993).

Hasted, J. B. (1973). Aqueous dielectrics, Chapman and Hall.

Huang, Y., et al. Measurements and models of electric fields in the in vivo human brain during transcranial electric stimulation. Elife 6, e18834 (2017).

Latikka, J., et al. Conductivity of living intracranial tissues. Physics in Medicine and Biology 46, 1611 (2001).

Ranck Jr, J. B. Specific impedance of rabbit cerebral cortex. Experimental neurology 7, 144-152 (1963).

Rigaud, B., et al. Tissue characterization by impedance: a multifrequency approach. Physiological Measurement 15, A13 (1994).

Stoy, R. D., et al. Dielectric properties of mammalian tissues from 0.1 to 100 MHz; a summary of recent data. Physics in Medicine and Biology 27, 501 (1982).

VAN, A. H., et al. Specific impedance of rabbit's cortical tissue. The American journal of physiology 205, 203-207 (1963).

Vöröslakos, M., et al. Direct effects of transcranial electric stimulation on brain circuits in rats and humans. Nature communications 9, 1-17 (2018).

Yamamoto, T., et al. Electrical properties of the epidermal stratum corneum. Medical and biological engineering 14, 151-158 (1976).
